# Differed IL-1 Beta Response between Active TB and LTBI Cases by Ex Vivo Stimulation of Human Monocyte-Derived Macrophage with TB-Specific Antigen

**DOI:** 10.1155/2019/7869576

**Published:** 2019-10-29

**Authors:** Meng-Rui Lee, Lih-Yu Chang, Chia-Hao Chang, Bo-Shiun Yan, Jann-Yuan Wang, Wan-Hsin Lin

**Affiliations:** ^1^Department of Internal Medicine, National Taiwan University Hospital, Hsin-Chu Branch, No. 25, Lane 442, Sec.1, Jingguo Rd., Hsinchu City 30059, Taiwan; ^2^Department of Internal Medicine, National Taiwan University Hospital, No. 7, Chung Shan S. Rd., Zhongzheng Dist., Taipei City 10002, Taiwan; ^3^Institute of Epidemiology and Preventive Medicine, College of Public Health, National Taiwan University, No. 1, Sec. 4, Roosevelt Rd., Taipei 10617, Taiwan; ^4^Institute of Biochemistry and Molecular Biology, National Taiwan University, College of Medicine, No. 1, Sec. 1, Jen Ai Rd., Zhongzheng Dist., Taipei City 10051, Taiwan

## Abstract

**Background:**

The difference of macrophage-specific interleukin-1 beta (IL-1b) response between latent tuberculosis infection (LTBI) and active tuberculosis (TB) remains less studied.

**Method:**

We performed this prospective study and recruited active TB patients, contacts with LTBI, and uninfected contacts. The gene and protein expression of human monocyte-derived macrophage (hMDM) after ex vivo stimulation by early secretory antigenic target-6KD (ESAT-6) and tuberculin purified protein derivatives (PPD) was studied by real-time PCR and flow cytometry. The effect of caspase-1 inhibitor was also studied.

**Result:**

The IL-1b gene expression after 6 hr ESAT-6 1 *μ*g/ml stimulation was different among active TB patients (*n* = 12), LTBI cases (*n* = 12), and uninfected contacts (*n* = 23) (log fold change: 0.98 ± 1.26 vs. 2.20 ± 0.96 vs. 2.20 ± 0.96, *P* = 0.013). The IL-1b gene expression at 24 hours was higher than that at 6 hours in LTBI cases (*n* = 4) and uninfected contacts (*n* = 6). After 24 hr ESAT-6 1 *μ*g/ml stimulation, the percentage of IL-1b-expressed hMDM was borderline lower in the active TB patients (*n* = 9) than in the LTBI cases (*n* = 10) (14.0 ± 11.2% vs. 31.6 ± 22.5%, *P* = 0.065). Compared with ESAT-6 1 *μ*g/ml stimulation but without the addition of caspase-1 inhibitor (CasI) (55.6 ± 16.3%), the percentage of IL-1b-positive hMDMs decreased after addition of CasI (50 *μ*g/ml CasI: 49.8 ± 18.2%, *P* = 0.078; 100 *μ*g/ml CasI: 46.6 ± 20.8%, *P* = 0.030; 150 *μ*g/ml CasI: 33.7 ± 15.5%, *P* = 0.016).

**Conclusions:**

This study revealed that macrophage-specific IL-1b response differed among different stages of *Mycobacterium tuberculosis* infection. The role of IL-1b and inflammasome in the process of LTBI progressing to active TB warrants further investigation.

## 1. Introduction

Tuberculosis (TB) remains an important global infectious disease with an estimated 10.4 million new cases and 1.4 million deaths in 2015 [1]. The World Health Organization (WHO) has set its goal in eradicating TB as a public health problem, aiming to achieve 50% reduction and 90% reduction in TB incidence by 2025 and 2035, respectively [2]. Latent tuberculosis infection (LTBI), which was underestimated to affect one-third of the overall human population, also emerged as an important target for better TB infection control [3]. To achieve the global goal to significantly reduce TB disease burden, a better understanding of the pathophysiology underlying the progression of LTBI to active TB was urgently needed.

Macrophages are primary host cells against *Mycobacterium tuberculosis* (*M. tb*) infection and are responsible for the regulation of various cytokines [4]. Macrophages are also involved in the initial infection process of *M. tb*, including being infected and granuloma formation response [5]. Macrophages are also the main cells for inflammasome expression, which were crucial for triggering the inflammation process and defending against intracellular pathogens [6, 7]. Due to sitting in a central position for controlling and preventing *M. tb* disease, macrophage and inflammasome functions have been intensely investigated [5]. Interleukin-1 beta (IL-1b) stands as the end product of inflammasome activation and plays a pleiotropic role in resistance to *M. tb* infection [4, 8].

LTBI refers to the status where viable *M. tb* is contained in a human and does not lead to clinical symptoms [9]. At the same time, persons with LTBI were at ineligible risk for progressing to active TB [3]. It would be important to explore the association between *M. tb*-induced macrophage-dependent host immune responses, particularly IL-1b and tumor necrosis factor-alpha (TNFa), and *M. tb* disease status. While abundant studies may exist in the literature regarding the role of IL-1b and TB, few studies have focused on the differential gene expression and regulation of macrophage-related IL-1b between LTBI and active TB. Most of them were either cell-line or animal studies, and only few studies have used human peripheral blood monocytes for experiment. We, therefore, conducted this study and investigated the macrophage inflammasomal responses with and without the caspase-1 inhibitor by gene and protein expression after ex vivo stimulation with TB antigens using real-time PCR and flow cytometry.

## 2. Materials and Methods

### 2.1. Study Design and Duration

This prospective study was conducted in the National Taiwan University Hospital from August 2015 to July 2017. All experiments were performed in accordance with relevant guidelines and regulations.

### 2.2. Study Population and Blood Sampling

We followed the definition of the study population and participant recruitment of Lee et al. [10]. We prospectively enrolled adult patients (age between 20 and 90 years) with culture- or histology-confirmed active TB (TB group). Close contacts of TB were defined as an exposure duration of 8 hours or more within one day or a cumulative duration of 40 hours or more without wearing adequate personal protective equipment for airborne transmission precautions. Close contacts of TB were then further screened for LTBI by using the QuantiFERON-TB Gold In-Tube (QFT) assay (Qiagen). If active TB was a concern, the contacts also received chest radiography and mycobacteriologic study (acid-fast smear and mycobacterial culture) from 3 sputum samples to exclude the possibility of active TB disease [11]. Uninfected contacts and LTBI contacts were considered for those with negative and positive QFT, respectively. After providing informed consent, they received peripheral blood sampling for further experiments.

The exclusion criteria were as follows: (1) human immunodeficiency virus infection, (2) bleeding tendency that increased the risk of blood sampling, and (3) life expectancies less than 6 months in TB patients or 2 years in household contacts.

### 2.3. QuantiFERON-TB Gold In-Tube (QFT®)

QFT was performed according to the manufacturer's instructions. The IFN-gamma level of the postreaction supernatant was then measured by an enzyme-linked immune-sorbent assay. Results were interpreted as positive, negative, or indeterminate accordingly [10].

### 2.4. Gene and Protein Expression of Human Monocyte-Derived Macrophages (hMDMs)

#### 2.4.1. Isolation of Peripheral Blood Mononuclear Cells (PBMCs)

We followed the PBMC isolation methods of Shu et al. [12]. Briefly, peripheral blood was sampled in a heparin-containing tube from enrolled participants. We used the Ficoll-Paque PLUS (GE Healthcare Life Sciences, Sweden) to isolate mononuclear cells. Mononuclear cells were then suspended in medium containing RPMI-1640 (Life Technologies; U.S.A.), 10% fetal bovine serum (FBS), and 1% penicillin/streptomycin (Life Technologies, USA). We then counted the viable cell by Scepter™ 2.0 Handheld Automated Cell Counter (Millipore Corporation, Billerica, MA, USA).

#### 2.4.2. Isolation and Differentiation of Peripheral Blood Monocytes

We isolated human monocytes from PBMCs by using a CD14-positive selection system (MACS system, Miltenyi Biotec Inc.), followed by cultivation in RPMI-1640 medium supplemented with 10% FBS, 50 mM 2-mercaptoethanol (Sigma-Aldrich, USA), and 50 ng/ml recombinant human macrophage colony-stimulating factor (R&D systems, USA) for 5 days to differentiate into macrophages. The culture medium was supplemented on the 3^rd^ day of differentiation. This process was also described previously [12].

#### 2.4.3. In Vitro Stimulation of hMDMs

The hMDMs were cultured for either 6 or 24 hours in the following six conditions: (1) no antigen, (2) early secretory antigenic target-6KD (ESAT-6) 1 *μ*g/ml (BEI Resources, Manassas, United States), (3) ESAT-6 5 *μ*g/ml, (4) tuberculin PPD 10 *μ*g/ml (BOVIGAM® Tuberculin PPD Stimulating Antigen, Life Technologies, USA), (5) tuberculin PPD 50 *μ*g/ml, and (6) lipopolysaccharide (LPS) 1 *μ*g/ml (Sigma-Aldrich, USA). The advantage and reason of stimulating with different antigen concentrations are that in addition to finding a potential dose response of hMDM stimulation to *M. tb* antigens, the optimal stimulating concentration of *M. tb* antigens remained unknown [13].

### 2.5. Real-Time PCR for Assessing Inflammasomal Response

#### 2.5.1. RNA Extraction

Total cellular RNA was extracted by using a Direct-zol™ RNA MiniPrep Kit (Zymo Research) according to the manufacturer's instructions. First-strand cDNA was synthesized using the iScript cDNA Synthesis kit (Life Science) as per the manufacturer's instructions.

#### 2.5.2. PCR Protocol

PCR reaction was performed using the iQ™ SYBR® Green Supermix (Bio-Rad) under the following conditions: PCR mixtures were denatured at 95°C for 3 minutes, followed by 39 cycles of 10 seconds at 95°C, 30 seconds at 58°C, 10 seconds at 95°C, and 5 seconds at 70°C for amplification. The mRNA expression levels of TNFa, IL-1b, mannose receptor (MR), and NLR Family Pyrin Domain Containing 3 (NLRP3) were normalized to their respective glyceraldehyde 3-phosphate dehydrogenase (GAPDH) expression. The sequences of sense and antisense primers are shown in Table 1 according to a previous report [14].

### 2.6. Flow Cytometry for Assessing IL-1b and TNFa Protein Expression

The hMDM was retrieved after 6 or 24 hours of in vitro stimulation in a medium containing 10% FBS and measured using flow cytometry (FACSVerse, BD Biosciences, USA). A protein transport inhibitor (GolgiStop™, BD Bioscience, USA) was added to the coculture. The retrieved cells were stained with anti-CD14-PerCP, anti-IL-1b-FITC, and anti-TNFa-PE. Data were analyzed using BD FACSuite V software (BD Biosciences, USA). We discriminated the cell population using forward scatter (FSC) and side scatter (SSC) and measured the percentage of IL-1b- and TNFa-expressed cells, respectively. We also calculated mean fluorescent intensity (MFI) in arithmetic means.

### 2.7. In Vitro Stimulation and the Addition of Caspase-1 Inhibitor

To assess the inflammasome response, the caspase-1 inhibitor (CasI) in three different concentrations was also added to hMDMs retrieved after 6 or 24 hours of in vitro stimulation. The percentage of IL-1b- and TNFa-expressed cells after in vitro stimulation and caspase-1 inhibition was also evaluated with flow cytometry.

### 2.8. Statistical Analysis

Nonparametric methods including the Kruskal-Wallis test, Mann-Whitney *U* test, and Wilcoxon signed-rank test tests were used for gene and protein expression comparison. All data analyses were performed using SAS version 9.4 (SAS Institute Inc., Cary, NC, USA). A *P* < 0.05 on a two-sided test was considered statistically significant.

## 3. Results

### 3.1. Inflammasome Response by Gene Expression

**Figure 1:**
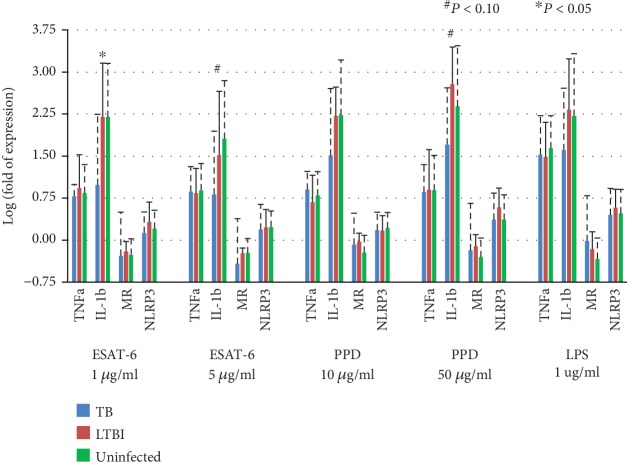
Inflammasomal gene expression in human monocyte-derived macrophages from 12 active tuberculosis (TB) patients, 12 latent TB infection (LTBI) cases, and 23 uninfected contacts after 6 hr antigen stimulation measured by real-time PCR. ESAT-6: early secretory antigenic target-6KD; IL-1b: interleukin-1 beta; LPS: lipopolysaccharide; MR: mannose receptor; NLRP3: NLR Family Pyrin Domain Containing 3; PPD: tuberculin purified protein derivative; TNFa: tumor necrosis factor-alpha.

A total of 47 subjects, including 12 active TB patients, 12 LTBI cases, and 23 uninfected contacts, were available for inflammasomal gene expression study after 6 hr antigen stimulation. Under ESAT-6 1 *μ*g/ml stimulation, the IL-1b gene log fold change was 0.98 ± 1.26 for active TB patients, 2.20 ± 0.96 for LTBI cases, and 2.20 ± 0.96 for uninfected contacts (*P* = 0.013). Under ESAT-6 5 *μ*g/ml stimulation, the IL-1b gene expression log fold change was 0.82 ± 1.13 for active TB patients, 1.52 ± 1.14 for LTBI cases, and 1.81 ± 1.04 for uninfected contacts (*P* = 0.057). Under PPD 50 *μ*g/ml stimulation, the IL-1b gene expression log fold change was 1.71 ± 1.01 for active TB patients, 2.78 ± 0.66 for LTBI cases, and 2.39 ± 1.08 for uninfected contacts (*P* = 0.091). The poststimulation IL-1b gene expression was always lowest in active TB patients among the three groups, whereas it was similar in the other two groups (Figure 1). Gene expression of TNFa, MR, and NLRP3 after 6 hr antigen stimulation was not statistically different between the three groups.

**Figure 2:**
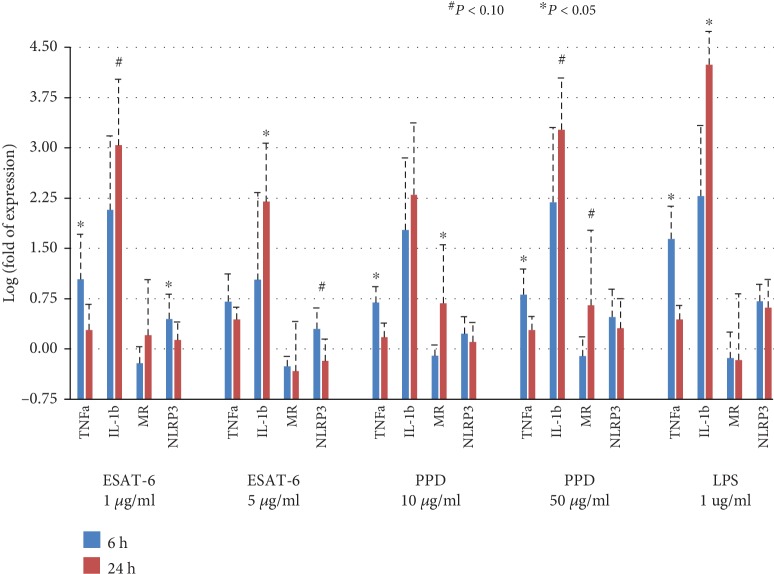
Inflammasomal gene expression in human monocyte-derived macrophages from 4 latent TB infection (LTBI) cases and 6 uninfected contacts after 6 hr and 24 hr antigen stimulations measured by real-time PCR. ESAT-6, early secretory antigenic target-6KD; IL-1b: interleukin-1 beta; LPS: lipopolysaccharide; MR: mannose receptor; NLRP3: NLR Family Pyrin Domain Containing 3; PPD: tuberculin purified protein derivative; TNFa: tumor necrosis factor-alpha.

Results of inflammasomal gene expression after 6 hr and 24 hr stimulations were both available in 4 of the 12 LTBI cases and 6 of the 23 uninfected contacts. Comparing between the 6 hr and 24 hr stimulations, IL-1b gene expression was significantly upregulated under ESAT-6 5 *μ*g/ml (log fold change 1.03 ± 1.30 vs. 2.20 ± 0.87, *P* = 0.039) and borderline upregulated under ESAT-6 1 *μ*g/ml (log fold change 2.08 ± 1.10 vs. 3.04 ± 0.99, *P* = 0.065) and tuberculin PPD 50 *μ*g/ml (log fold change 2.20 ± 1.12 vs. 3.27 ± 0.77, *P* = 0.078) (Figure 2). On the contrary, TNFa gene expression was significantly downregulated under ESAT-6 1 *μ*g/ml (log fold change 1.04 ± 0.67 vs. 0.28 ± 0.39, *P* = 0.004), tuberculin PPD 10 *μ*g/ml (log fold change 0.69 ± 0.24 vs. 0.17 ± 0.21, *P* = 0.003, *P* = 0.008), and tuberculin PPD 50 *μ*g/ml (log fold change 0.81 ± 0.38 vs. 0.28 ± 0.20, *P* = 0.008) after 24 hr stimulation comparing with that after 6-hour stimulation. The NLRP3 gene expression was borderline downregulated under ESAT-6 1 *μ*g/ml (log fold change 0.45 ± 0.37 at 6 hours vs. 0.13 ± 0.27 at 24 hours, *P* = 0.065).

### 3.2. IL-1b and TNFa Protein Expression

**Figure 3:**
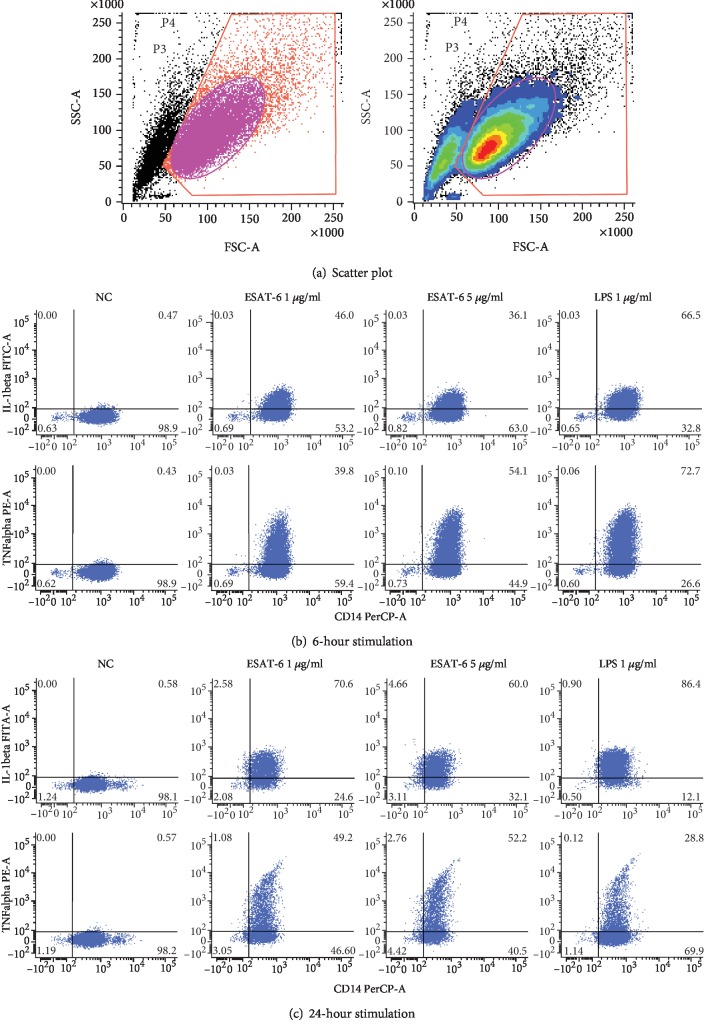
Representative illustration of scatter plot (a), 6 hr stimulation (b), and 24 hr stimulation (c) in the flow cytometry study. ESAT-6: early secretory antigenic targe-6KD; IL: interleukin; LPS: lipopolysaccharide; NC: negative control; TNF: tumor necrosis factor.

**Figure 4:**
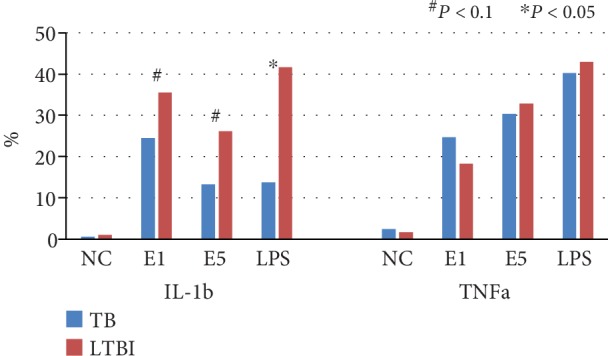
Interleukin-1 beta (IL-1b) and tumor necrosis factor-alpha (TNFa) responses of human monocyte-derived macrophages in active tuberculosis (TB) patients and latent TB infection (LTBI) cases after TB-specific antigen stimulation for 6 hours by flow cytometry. E1: early secretory antigenic target-6KD (ESAT-6) 1 *μ*g/ml; E5: ESAT-6 5 *μ*g/ml; LPS: lipopolysaccharide 1 *μ*g/ml.

Representative illustration of flow cytometry results is shown in Figure 3. A total of 11 active TB patients and 17 LTBI cases were available for analysis of IL-1b and TNFa response of hMDMs after 6 hr TB-specific antigen stimulation. The percentage of IL-1b-positive hMDMs was borderline different between active TB patients and LTBI cases after 6 hr ESAT-6 1 *μ*g/ml (24.5 ± 15.7% vs. 35.5 ± 15.3%, *P* = 0.093) and ESAT-6 5 *μ*g/ml (13.3 ± 11.4% vs. 26.1 ± 12.1%, *P* = 0.073) stimulation, and was significantly less in active TB patients after LPS 1 *μ*g/ml stimulation (13.8 ± 14.7% vs. 41.6 ± 13.6%, *P* < 0.001) (Figure 4). The percentage of TNFa-positive hMDMs was not different between active TB patients and LTBI cases after ESAT-6 1 *μ*g/ml (24.6 ± 16.2% vs. 18.2 ± 10.7%, *P* = 0.404), ESAT-6 5 *μ*g/ml (30.3 ± 11.8% vs. 32.9 ± 12.6%, *P* = 0.805), and LPS 1 *μ*g/ml (40.2 ± 22.7% vs. 42.9 ± 23.3%, *P* = 0.644) stimulations.

**Figure 5:**
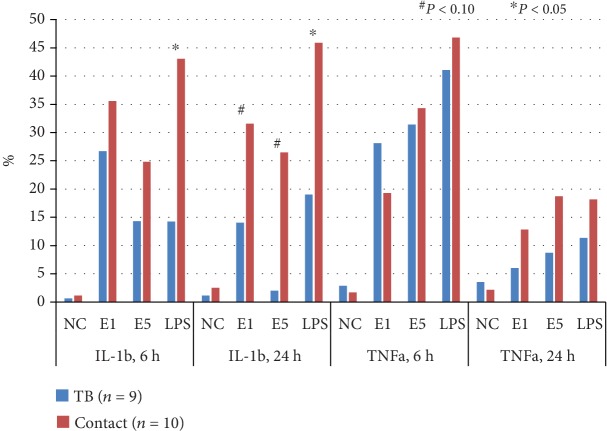
Interleukin-1 beta (IL-1b) and tumor necrosis factor-alpha (TNFa) response of human monocyte-derived macrophages in active tuberculosis (TB) patients and latent TB infection (LTBI) cases after TB-specific antigen stimulation for 6 and 24 hours by flow cytometry. E1: early secretory antigenic target-6KD (ESAT-6) 1 *μ*g/ml; E5: ESAT-6 5 *μ*g/ml; LPS: lipopolysaccharide 1 *μ*g/ml.

Results of cytokine responses after 6 hr and 24 hr stimulations were both available in 9 of the 11 active TB patients and 10 of the 17 LTBI cases. The percentage of IL-1b-positive hMDMs was borderline significantly less in active TB patients than in LTBI cases after ESAT-6 1 *μ*g/ml (14.0 ± 11.2% vs. 31.6 ± 22.5%, *P* = 0.065) and 5 *μ*g/ml (2.0 ± 2.4% vs. 26.5 ± 29.6%, *P* = 0.064) stimulation for 24 hours. The percentage of IL-1b-positive hMDMs was similar after 6 hr and 24 hr stimulations by ESAT-6 1 *μ*g/ml (35.6 ± 12.4% vs. 31.6 ± 22.5%, *P* = 0.695) and ESAT-6 5 *μ*g/ml (24.8 ± 13.1 vs. 26.5 ± 29.6%, *P* > 0.999) in LTBI cases, whereas it was significantly decreased by ESAT-6 1 *μ*g/ml stimulation in active TB patients (26.7 ± 16.6 vs. 14.0 ± 11.2, *P* = 0.012). For TNFa-positive hMDM percentage, no difference was observed among ESAT-6 1 *μ*g/ml, ESAT-6 5 *μ*g/ml, and LPS 1 *μ*g/ml stimulations between active TB patients and LTBI cases (Figure 5).

In MFI analysis, the MFI of IL-1b-positive hMDMs among TB cases was borderline lower than that among LTBI cases (64.1 ± 13.9 vs. 83.8 ± 20.0, *P* = 0.075) after 6 hr stimulation by ESAT-6 5 *μ*g/ml. Also, there was a tendency for lower MFI of IL-1b-positive hMDMs among TB patients than among LTBI contacts after 24 hr stimulation by ESAT-6 1 *μ*g/ml (64.0 ± 19.6 vs. 89.8 ± 44.9, *P* = 0.124) and ESAT-6 5 *μ*g/ml (36.5 ± 9.9 vs. 84.0 ± 62.1, *P* = 0.163).

### 3.3. Caspase-1 Inhibition

Seven LTBI contacts were available for assessment of IL-1b-positive hMDMs after caspase-1 inhibition. Compared with ESAT-6 1 *μ*g/ml stimulation but without addition of CasI (55.6 ± 16.3%), the percentage of IL-1b-positive hMDMs decreased after addition of CasI (50 *μ*g/ml CI: 49.8 ± 18.2, *P* = 0.078; 100 *μ*g/ml CI: 46.6 ± 20.8, *P* = 0.030; 150 *μ*g/ml CI: 33.7 ± 15.5, *P* = 0.016). Compared with ESAT-6 1 *μ*g/ml stimulation but without addition of CasI (55.1 ± 9.1), the percentage of TNFa-positive hMDMs was not different after ESAT-6 1 *μ*g/ml stimulation and caspase-1 inhibition (50 *μ*g/ml CasI: 57.8 ± 15.5, *P* = 0.406, 100 *μ*g/ml CasI: 55.3 ± 12.1, *P* > 0.999; 150 *μ*g/ml CasI: 53.8 ± 11.2, *P* = 0.578).

## 4. Discussion

By ex vivo stimulation of hMDMs from different stages of *M. tb* infection, we found that IL-1b was significantly highly expressed in the LTBI cases and uninfected contacts as compared to the active TB patients. On the contrary to TNFa, the expression of IL-1b was accentuated when the stimulation was prolonged from 6 hours to 24 hours. The flow cytometry study revealed similar findings, showing that the percentage of IL-1b-positive hMDMs was markedly elevated in LTBI cases, whereas the percentage of TNFa-positive hMDMs was not different between LTBI cases and active TB patients. Applying the caspase-1 inhibitor decreased the percentage of IL-1b-positive hMDMs. Our findings highlight the role of IL-1b and inflammasome in TB pathogenesis, especially in the progression of LTBI to active TB.

Most studies published regarding TB and IL-1b mainly focused on the comparison between active TB and uninfected or between pretreated and posttreated statuses [15]. Studies that have investigated the role of IL-1b among LTBI patients often measure the systemic IL-1b level [16, 17]. Furthermore, most studies used a mouse model rather than peripheral blood monocyte-derived macrophages from human active TB and LTBI patients [4, 18]. The uniqueness of our study, therefore, lies in studying macrophage-specific IL-1b expression at gene and protein levels and, more importantly, targeting active TB patients as well as LTB cases. Indeed, the focus of recent studies has been in exploring the interaction of macrophages and latent tuberculosis infection. For instance, a recent study showed that compared with active TB, antibodies production from latent tuberculosis leads to enhanced inflammasomal activation and macrophage killing of intracellular *M. tb* [19]. Given that LTBI infection is often the first step of developing active TB, elucidating LTBI pathophysiology is therefore important [20].

IL-1b is a double-edged sword in TB disease, with its antimicrobial effect as a protective role for disease development while prolonged expression could lead to progressive tissue damage [21]. In an animal model, IL-1 receptor knockout mice lose early pathogen control following aerogenic infection with *M. tb* [22]. Our study revealed that IL-1b gene expression after stimulation was upregulated in both TB patients and LTBI cases while the degree of elevation was more obvious in the latter group. Also, the percentage of IL-1b-positive macrophages also decrease substantially following 24 hr stimulation compared with the 6 hr stimulation in active TB patients. This finding could have two implications. First, the high IL-1b response by macrophages in the LTBI status may indicate activation of immunity to prevent development to active TB. The relatively low response among active TB patients may indicate a defective immunity response to TB bacilli and this would contribute to the development of active disease. Interestingly and contradictorily, a previous study has discovered a high IL-1b expressing genotype, which was associated with the development of active tuberculosis, higher disease severity, and poorer treatment outcome in TB patients [21]. On the other hand, the relatively low level of IL-1b response may also be a human regulatory mechanism to avoid further tissue inflammation and destruction [23–25]. Second, our study revealed that IL-1b could be the target for preventing progression from LTBI to active TB. Enhancing IL-1b activity may be an option to halt TB infection progression [26]. Whether use of IL-1b antagonists would increase the risk of LTBI progression to active TB also needs to be investigated [27, 28].

The secretion of IL-1b by macrophages comes from two pathways, including the inflammasome and NF-*κ*B pathways [4]. NF-*κ*B was known to mediate and enhance the secretion of various cytokines, including IL-1b and TNFa [29]. In our study, TNFa secretion was not elevated and this may indicate that the higher response of IL-1b by macrophages was most likely through the pathway of inflammasomes. Furthermore, caspase-1 is an important inflammasome activator, and the findings of decreased IL-1b-positive hMDMs after addition of the caspase-1 inhibitor further supported the contribution of inflammasomes. Though our mRNA gene expression profile only revealed transient increase in expression of the NLRP3 pathway, our study still highlights the importance of inflammasomes in TB pathogenesis, through the effect of IL-1b. NLRP3 is an important inflammasome sensor, and study has shown that NLRP3 gene polymorphism is associated with increased IL-1b production and better *M. tb* control [30]. The activation of NLRP3 by mycobacteria was more complex, and several components including ESAT-6 secretion system (ESX)-1, the never in mitosis A-related kinase 7 (Nek7), were considered important activators of NLRP3 [31, 32]. The interaction and association between inflammasomes, activating components, and IL-1b production were less clear and remained to be studied.

Our study also has important implications for clinical practice. Due to limited performance of current biomarkers for monitoring TB treatment response and disease status, newer and more sophisticated biomarkers are needed [33]. Recently, Adekambi et al. found that host blood-based biomarkers including CD38, HLA-DR, and Ki-67 on *M. tb*-specific CD4^+^ T cells could help discriminate between active TB and LTBI and could be a biomarker for monitoring treatment response and cure [34]. Furthermore, combined analysis of *M. tb*-specific CD4 and CD8 T cell responses has also been proposed to be a powerful diagnostic tool for diagnosing TB [35]. Besides *M. tb*-specific T cell immune markers, studies targeting the ratio of the myeloid over lymphoid cells also highlight the importance of circulating monocytes as an important of biomarker for predicting development of active TB among contacts [36, 37]. Also, antigen-specific IL-1b can be used as a biomarker for differential diagnosis of pulmonary TB and LTBI [38]. Interestingly, by measuring cytokines in supernatants generated from QFT tubes, higher IL-1b and TNFa levels were observed in active TB patients than in contacts and uninfected controls [38]. Additionally, our findings add knowledge to the current understanding of TB immunology and suggested that IL-1b-positive hMDM could be a novel immune cell signature biomarker differentiating between LTBI cases and active TB patients.

Our study also has limitations. Large and sufficient amount of blood specimens from participants were hard to collect in the clinical setting, and this may cause inconsistent number of cases measuring sequential change of IL-1b response. This, however, may reflect the distinguishing feature (using peripheral blood monocytes from active TB and LTBI patients) of our study. Second, different immunocompromised statuses can alter immune response and are not readily excluded in our study. Diabetes, for instance, is an important disease contributing to tuberculosis and can affect the measured parameters and is not among our exclusion criteria [39, 40]. Third, we did not check genetic polymorphisms of targeted cytokines and inflammasomes [41, 42]. The association between corresponding genetic polymorphisms and TB disease course was worth discussing and could be the perspective of future studies [43].

In conclusion, we found that macrophage-specific IL-1b response differed between LTBI cases and active TB patients after ex vivo TB-specific antigen stimulation, while response in LTBI was higher than active TB patients. In active TB patients, the IL-1b-positive human monocyte-derived macrophage after stimulation was decreased compared with LTBI cases. IL-1b gene expression was also decreased in active TB patients compared with LTBI cases. Our results suggested that IL-1b plays a role in the progression of LTBI patients, and further studies regarding mechanism and clinical implications were warranted.

## Figures and Tables

**Table 1:** Sequences of sense and antisense primers in real-time PCR.

Primer	Sense (5′-3′)	Antisense (5′-3′)
GAPDH	CCTCAAGATCATCAGCAATG	CACGATACCAAAGTTGTCAT
TNFa	ACAAGCCTGTAGCCCATGTT	AAAGTAGACCTGCCCAGACT
IL-1b	GGATATGGAGCAACAAGTGG	GAAGTCAGTTATATCCTGGC
MR	GTCATCATTGTGATCCTCCTG	GATGACCGAGTGTTCATTCTG
NLRP3	CCAAGAATCCACAGTGTAACC	CTTCACAGAACATCATGACCC

GAPDH: glyceraldehyde 3-phosphate dehydrogenase; IL-1b: interleukin-1 beta; MR: mannose receptor; NLRP3: NLR Family Pyrin Domain Containing 3; TNFa: tumor necrosis factor-alpha.

## Data Availability

The data will be made available from the corresponding author upon reasonable request.

## Abbreviations

CasI: Caspase-1 inhibitor
hMDM: Human monocyte-derived macrophages
IL-1b: Interleukin-1b
*M. tb*: Mycobacterium tuberculosis
LTBI: Latent tuberculosis infection
QFT assay: QuantiFERON-TB Gold In-Tube assay
TB: Tuberculosis
TNFa: Tumor necrosis factor-alpha.

## References

[1] World Health Organization (2016). *Global Tuberculosis Report 2016*.

[2] World Health Organization (2014). *Global Strategy and Targets for Tuberculosis Prevention, Care and Control after 2015*.

[3] Getahun H., Matteelli A., Chaisson R. E., Raviglione M. (2015). Latent mycobacterium tuberculosis infection. *The New England Journal of Medicine*.

[4] Krishnan N., Robertson B. D., Thwaites G. (2013). Pathways of IL-1*β* secretion by macrophages infected with clinical *Mycobacterium tuberculosis* strains. *Tuberculosis*.

[5] Huynh K. K., Joshi S. A., Brown E. J. (2011). A delicate dance: host response to mycobacteria. *Current Opinion in Immunology*.

[6] Awad F., Assrawi E., Jumeau C. (2017). Impact of human monocyte and macrophage polarization on NLR expression and NLRP3 inflammasome activation. *PLoS One*.

[7] Weiss G., Schaible U. E. (2015). Macrophage defense mechanisms against intracellular bacteria. *Immunological Reviews*.

[8] Mayer-Barber K. D., Andrade B. B., Oland S. D. (2014). Host-directed therapy of tuberculosis based on interleukin-1 and type I interferon crosstalk. *Nature*.

[9] Cadena A. M., Fortune S. M., Flynn J. L. (2017). Heterogeneity in tuberculosis. *Nature Reviews Immunology*.

[10] Lee M. R., Chang C. H., Chang L. Y. (2019). CD8 response measured by QuantiFERON-TB Gold Plus and tuberculosis disease status. *The Journal of Infection*.

[11] Luh K. (2015). Taiwan guidelines for TB diagnosis and treatment.

[12] Shu C.-C., Wang J.-Y., Wu M.-F. (2017). Attenuation of lymphocyte immune responses during Mycobacterium avium complex-induced lung disease due to increasing expression of programmed death-1 on lymphocytes. *Scientific Reports*.

[13] Giampietro F., de Waard J. H., Rivas-Santiago B., Enciso-Moreno J. A., Salgado A., Araujo Z. (2010). In vitro levels of cytokines in response to purified protein derivative (PPD) antigen in a population with high prevalence of pulmonary tuberculosis. *Human Immunology*.

[14] Wu M. F., Chen S. T., Yang A. H. (2013). CLEC5A is critical for dengue virus-induced inflammasome activation in human macrophages. *Blood*.

[15] Kathamuthu G. R., Moideen K., Bhaskaran D. (2017). Reduced systemic and mycobacterial antigen-stimulated concentrations of IL-1*β* and IL-18 in tuberculous lymphadenitis. *Cytokine*.

[16] Anuradha R., Munisankar S., Bhootra Y. (2016). Coexistent malnutrition is associated with perturbations in systemic and antigen-specific cytokine responses in latent tuberculosis infection. *Clinical and Vaccine Immunology*.

[17] Kumar N. P., George P. J., Kumaran P., Dolla C. K., Nutman T. B., Babu S. (2014). Diminished systemic and antigen-specific type 1, type 17, and other proinflammatory cytokines in diabetic and prediabetic individuals with latent *Mycobacterium tuberculosis* infection. *The Journal of Infectious Diseases*.

[18] Marinho F. V., Fahel J. S., Scanga C. A. (2016). Lack of IL-1 receptor-associated kinase-4 leads to defective Th1 cell responses and renders mice susceptible to mycobacterial infection. *The Journal of Immunology*.

[19] Lu L. L., Chung A. W., Rosebrock T. R. (2016). A functional role for antibodies in tuberculosis. *Cell*.

[20] Lee M. R., Huang Y. P., Kuo Y. T. (2017). Diabetes mellitus and latent tuberculosis infection: a systemic review and metaanalysis. *Clinical Infectious Diseases*.

[21] Zhang G., Zhou B., Li S. (2014). Allele-specific induction of IL-1*β* expression by C/EBP*β* and PU.1 contributes to increased tuberculosis susceptibility. *PLoS Pathogens*.

[22] Fremond C. M., Togbe D., Doz E. (2007). IL-1 receptor-mediated signal is an essential component of MyD88-dependent innate response to *Mycobacterium tuberculosis* infection. *The Journal of Immunology*.

[23] Kanarek N., Grivennikov S. I., Leshets M. (2014). Critical role for IL-1*β* in DNA damage-induced mucositis. *Proceedings of the National Academy of Sciences of the United States of America*.

[24] Chao W.-C., Yen C.-L., Hsieh C.-Y. (2017). Mycobacterial infection induces higher interleukin-1*β* and dysregulated lung inflammation in mice with defective leukocyte NADPH oxidase. *PLoS One*.

[25] Pan T., Shi X., Chen H. (2018). Geniposide suppresses interleukin-1*β*-induced inflammation and apoptosis in rat chondrocytes via the PI3K/Akt/NF-*κ*B signaling pathway. *Inflammation*.

[26] Sharma M., Mohapatra J., Acharya A., Deshpande S. S., Chatterjee A., Jain M. R. (2013). Blockade of tumor necrosis factor-*α* converting enzyme (TACE) enhances IL-1*β* and IFN-*γ* via caspase-1 activation: a probable cause for loss of efficacy of TACE inhibitors in humans?. *European Journal of Pharmacology*.

[27] Lopalco G., Rigante D., Giannini M. (2016). Safety profile of anakinra in the management of rheumatologic, metabolic and autoinflammatory disorders. *Clinical and Experimental Rheumatology*.

[28] Giampietro C., Fautrel B. (2012). Anti-interleukin-1 agents in adult onset Still's disease. *International Journal of Inflammation*.

[29] Tak P. P., Firestein G. S. (2001). NF-*κ*B: a key role in inflammatory diseases. *The Journal of Clinical Investigation*.

[30] Eklund D., Welin A., Andersson H. (2014). Human gene variants linked to enhanced NLRP3 activity limit intramacrophage growth of *Mycobacterium tuberculosis*. *The Journal of Infectious Diseases*.

[31] Mishra B. B., Moura-Alves P., Sonawane A. (2010). *Mycobacterium tuberculosis* protein ESAT-6 is a potent activator of the NLRP3/ASC inflammasome. *Cellular Microbiology*.

[32] Shi H., Wang Y., Li X. (2016). NLRP3 activation and mitosis are mutually exclusive events coordinated by NEK7, a new inflammasome component. *Nature Immunology*.

[33] Wallis R. S., Kim P., Cole S. (2013). Tuberculosis biomarkers discovery: developments, needs, and challenges. *The Lancet Infectious Diseases*.

[34] Adekambi T., Ibegbu C. C., Cagle S. (2015). Biomarkers on patient T cells diagnose active tuberculosis and monitor treatment response. *The Journal of Clinical Investigation*.

[35] Rozot V., Patrizia A., Vigano S. (2015). Combined use of *Mycobacterium tuberculosis*-specific CD4 and CD8 T-cell responses is a powerful diagnostic tool of active tuberculosis. *Clinical Infectious Diseases*.

[36] Naranbhai V., Hill A. V., Abdool Karim S. S. (2014). Ratio of monocytes to lymphocytes in peripheral blood identifies adults at risk of incident tuberculosis among HIV-infected adults initiating antiretroviral therapy. *The Journal of Infectious Diseases*.

[37] Naranbhai V., Fletcher H. A., Tanner R. (2015). Distinct transcriptional and anti-mycobacterial profiles of peripheral blood monocytes dependent on the ratio of monocytes: lymphocytes. *eBioMedicine*.

[38] Prabhavathi M., Kabeer B. S., Deenadayalan A., Raja A. (2015). Role of QuantiFERON-TB Gold antigen-specific IL-1*β* in diagnosis of active tuberculosis. *Medical Microbiology and Immunology*.

[39] Lee M. R., Huang Y. P., Kuo Y. T. (2017). Diabetes mellitus and latent tuberculosis infection: a systematic review and metaanalysis. *Clinical Infectious Diseases*.

[40] Kumar Nathella P., Babu S. (2017). Influence of diabetes mellitus on immunity to human tuberculosis. *Immunology*.

[41] Souza de Lima D., Ogusku M. M., Sadahiro A., Pontillo A. (2016). Inflammasome genetics contributes to the development and control of active pulmonary tuberculosis. *Infection, Genetics and Evolution*.

[42] Pontillo A., Carvalho M. S., Kamada A. J. (2013). Susceptibility to *Mycobacterium tuberculosis* infection in HIV-positive patients is associated with CARD8 genetic variant. *Journal of Acquired Immune Deficiency Syndromes*.

[43] Hall N. B., Igo R. P., Malone L. L. (2015). Polymorphisms in *TICAM2* and *IL1B* are associated with TB. *Genes and Immunity*.

